# Anticarcinogenic impact of extracellular vesicles (exosomes) from cord blood stem cells in malignant melanoma: A potential biological treatment

**DOI:** 10.1111/jcmm.17639

**Published:** 2022-12-22

**Authors:** Parisa Naeem, Adi Baumgartner, Nader Ghaderi, Farshid Sefat, Maysa Alhawamdeh, Saeed Heidari, Fanila Shahzad, Karthic Swaminathan, Pouria Akhbari, Mohammad Isreb, Diana Anderson, Andrew Wright, Mojgan Najafzadeh

**Affiliations:** ^1^ School of Life Sciences University of Bradford Bradford UK; ^2^ School of Science, Technology and Health, Biosciences York St John University York UK; ^3^ Bradford Teaching Hospitals NHS Foundation Trust St Luke's Hospital Bradford UK; ^4^ Department of Biomedical and Electronics Engineering, Faculty of Engineering and Informatics University of Bradford Bradford UK; ^5^ Department of Medical Laboratory Sciences, Faculty of Allied Medical Sciences Mutah University Alkarak Jordan; ^6^ Cell Therapy and Tissue engineering Department, Faculty of Medical Sciences Shahid Beheshti University Tehran Iran; ^7^ Department of Biosciences Durham University Durham UK; ^8^ Institute of Biomedical and Clinical Science, College of Medicine and Health University of Exeter Exeter UK; ^9^ School of Pharmacy and Medical Sciences University of Bradford Bradford UK

**Keywords:** anti‐mutagenic, biologic treatment, cord blood stem cells, exosomes, malignant melanoma

## Abstract

Incidence of Malignant Melanoma has become the 5th in the UK. To date, the major anticancer therapeutics include cell therapy, immunotherapy, gene therapy and nanotechnology‐based strategies. Recently, extracellular vesicles, especially exosomes, have been highlighted for their therapeutic benefits in numerous chronic diseases. Exosomes display multifunctional properties, including inhibition of cancer cell proliferation and initiation of apoptosis. In the present in vitro study, the antitumour effect of cord blood stem cell (CBSC)‐derived exosomes was confirmed by the CCK‐8 assay (*p* < 0.05) on CHL‐1 melanoma cells and improve the repair mechanism on lymphocytes from melanoma patients. Importantly, no significant effect was observed in healthy lymphocytes when treated with the exosome concentrations at 24, 48 and 72 h. Comet assay results (OTM and %Tail DNA) demonstrated that the optimal exosome concentration showed a significant impact (*p* < 0.05) in lymphocytes from melanoma patients whilst causing no significant DNA damage in lymphocytes of healthy volunteers was 300 μg/ml. Similarly, the Comet assay results depicted significant DNA damage in a melanoma cell line (CHL‐1 cells) treated with CBSC‐derived exosomes, both the cytotoxicity of CHL‐1 cells treated with CBSC‐derived exosomes exhibited a significant time‐dependent decrease in cell survival. Sequencing analysis of CBSC exosomes showed the presence of the let‐7 family of miRNAs, including let‐7a‐5p, let‐7b‐5p, let‐7c‐5p, let‐7d‐3p, let‐7d‐5p and two novel miRNAs. The potency of CBSC exosomes in inhibiting cancer progression in lymphocytes from melanoma patients and CHL‐1 cells whilst causing no harm to the healthy lymphocytes makes it a potential candidate as an anticancer therapy.

## 1 INTRODUCTION

Cancer is a global health issue associated with numerous complications.^1^ Research has highlighted various approaches that proved promising in the treatment of cancer. These include cell therapy,^2^, ^3^ immunotherapy,^4^ gene therapy^5^ and methods based on nanotechnology. These therapeutic approaches mainly focus on the inhibition of carcinogenesis by apoptosis and stimulation of the immune response against tumour cells. In the last few years, the ability of membrane‐bound extracellular vesicles (EVs) to mediate cellular communication has been extensively investigated. Multiple EVs have proved beneficial in the treatment of various chronic diseases, depending on their pathogenesis and aetiology. EVs are classified as microvesicles, exosomes and apoptotic bodies based on their biogenesis – with exosomes being most widely explored in regenerative research due to their therapeutic abilities.^6^ These nanosized particles range 30–150 nm in diameter and are secreted by most cell types exhibiting various cellular compartments.^7^ As an improvement in drug delivery, the EVs could be applied as drug nanocarriers, to generate nEV‐like cell‐derived nanovesicles (CNV) and enhance bioavailability.^8^ Unlike microvesicles, which develop by the budding of the plasma membrane, exosomes are formed by the fusion of the plasma membrane and multi‐vesicular bodies via the endo‐lysosomal pathway.^9^ The membrane of extracellular vesicles forms a lipid‐bilayer enriched with sphingomyelin, ceramide and cholesterol, ensuring high stability^10^ when hauling nucleic acids and proteins as their cargo between cells. These nanosized vesicles benefit from retaining their stability and biological function even after being stored at −80°C for up to 2 years.^11^

Mesenchymal stem cells (MSCs) are considered the most naïve cells exhibiting self‐renewal abilities. MSCs possess the potential to differentiate into highly specialized cells under certain cues.^12^ Research suggests that exosomes isolated from MSCs could act as transferors of biomarkers that could facilitate diagnostic examinations.^13^ MSCs have been highlighted to promote therapeutic interventions in clinical and pre‐clinical studies.^14^ The mechanism of action of these cells depends upon their origin, pluripotency and ability to proliferate. A large body of evidence suggests that the trophic effects of MSCs play a vital role in regenerative medicine, and their paracrine impact depends on the exosomes released from them.^13^ Previous studies demonstrated that human cord blood‐derived MSCs alleviated inflammatory diseases and focal cerebral ischaemia.^15^ Also, the use of mRNA lipid nanoparticle (LNP) as a form of vaccine was considered in SARS‐CoV‐2 vaccine development. To improve bioavailability and effectiveness of drug delivery, the LNP (mRNA and protein) was transformed into inhaled Lung‐Exo.^16^ Moreover, it was recently reported that human embryonic stem cells could reverse the tumorigenic microenvironment of melanoma cells to a less malignant phenotype. The authors, however, indicated that the inhibition of tumourigenicity in these cell lines was carried out by repressing cancer cell proliferation and triggering apoptosis.^17^

MicroRNAs (miRNAs) belong to a group of non‐coding RNAs (ncRNAs) responsible for regulating genes that attach to the 3′ UTR of their target messenger RNAs (mRNA), ultimately causing mRNA degradation.^18^ The cargo of exosomes is also reported to constitute miRNAs, other ncRNAs and mRNA but also cytoplasmic and lipid‐containing proteins, and other cellular components such as protein receptors, histocompatibility complex components, tetraspanins, PDCD6IP, TSG101 and tumour susceptibility gene proteins.^19^ In source cells, this comprehensive range of molecular cargo is sorted into exosomes by the Endosomal Sorting Complex Required for Transport (ESCRT).^19^ This feature of exosomes makes them a widely investigated subtype of extracellular vesicles compared to their countertypes. Moreover, in a study to treat malignant melanoma or in metastatic states, sonodynamic therapy (SDT) was introduced as loaded docetaxel, combining SDT and chemotherapy with redox/enzyme/ultrasound‐responsive chondroitin sulphate‐chlorine e6‐lipoic acid nanoplatforms to inhibit cell proliferation and migration in melanoma.^20^

Abnormal expression of miRNAs is associated with various types of cancer, including cancers of the prostate, breast and lung.^21^, ^22^ The *let‐7* miRNA, the first known human miRNA, was initially discovered in *Caenorhabditis elegans*.^23^ It was reported that this family of miRNA was responsible for regulating the timely division and differentiation of stem cells^24^ – with the main well‐established function of the let‐7 mi RNA family being the suppression of tumour growth and progression. In colon cancer, let‐7 is reported to suppress the expression of oncogenes *RAS* and *MYC*, thus inhibiting its progression.^25^ Moreover, it has also been suggested that let‐7 could inhibit the proliferation and growth of lung cancer cells in vivo and *vitro*.^26^ In prostate cancer, however, let‐7c‐5p, a subtype of miRNA let‐7c, is downregulated, ultimately leading to cancer cell migration and growth.^27^

In this study, the cargo of cord blood‐derived exosomes was hypothesized to comprise certain factors that may be able to inhibit tumour development by inducing apoptosis in cancer cells. To test this hypothesis, the cytotoxic effect of CBSC‐derived exosomes on melanoma cells (CHL‐1), lymphocytes of malignant melanoma and lymphocytes from healthy individuals were investigated. The Comet assay was conducted to evaluate the DNA damage in both CHL‐1 cells and lymphocytes from melanoma patients and healthy individuals after treatment with varying concentrations of cord blood stem cell (CBSC)‐derived exosomes.

## 2 MATERIALS AND METHODS

### 2.1 Preparation of human unrestricted somatic stem cells (USSCs)

The human USSCs were isolated from normal human cord blood provided by the obstetrics and gynaecology ward, Shariati hospital, Tehran‐Iran. Cord blood was collected from the mother and informed written consent was obtained. The mononuclear cells were obtained by Ficoll gradient separation (Biochrom) followed by RBCs lysis using ammonium chloride. Cells were cultured in T25 culture flasks (Costar) at a confluency of 5–7 × 10^6^ cells/ml. Cells were then cultured in myelocult medium (StemCell Technologies), low glucose DMEM, Glutamax (Thermo Fischer), Penicillin–Streptomycin‐Glutamine (100×, Thermo Fischer) and incubated at 37°C in 5% CO_2_ in a humidified atmosphere. Cultured cells were later detached when they reached 80% confluency using 0.25% trypsin.

### 2.2 Isolation of cord blood stem cells (CBCS)‐derived exosomes

The cultured media of human USSCs was collected and centrifuged at 300 *g* for 10 min, followed by 2000 *g* for 10 min and finally 10,000 *g* for 30 min. The supernatant was then collected and ultracentrifuged at 110,000 *g* for 70 min using a W32Ti rotor (L‐80 XP, Beckman Coulter). The pellet obtained was washed in phosphate buffer saline (PBS) and later centrifuged at 110,000 *g* for 70 min. Next, PBS was removed, and the exosomes were re‐suspended in 100 μl of PBS and nuclease‐free water. The exosomes were finally stored at −80°C freezer.

### 2.3 Transmission electron microscopy imaging

The morphology of exosomes was evaluated by transmission electron microscopy. Briefly, CBSC‐derived exosomes were fixed in 2% acetate and placed onto a copper grid, stained with 2% uranium dioxide acetate and later dried at room temperature for a few minutes. The samples were finally analysed at a magnification of 250,000×.

### 2.4 Ethical approval for sample collection

Blood samples were collected from three healthy volunteers and three melanoma patients, and ethical approval was granted by the University of Bradford subcommittee for ethics in research involving Human Subjects (Ref: 0405/8), and the Research Support and Governance office, Bradford Teaching Hospitals, NHS Foundation (Ref: RE DA 1202). Human UCB‐MSCs exosomes were a gift from our collaborators in Tehran's Taleghani Hospital. All the samples were collected from healthy mothers after obtaining consent. The cord blood samples were received from Tissue Bank at the University of Bradford (Ref: ET‐17‐088).

### 2.5 Cell culture

CHL‐1 cells were cultured in RPMI‐1640 medium (Sigma Aldrich) supplemented with 10% foetal bovine serum (Thermo Fischer) and 50 U/ml of a penicillin/streptomycin mixture (Sigma Aldrich). The cells were grown in 75 cm^2^ flasks (Corning Inc.) and incubated at 37°C with 5% CO^2^.

### 2.6 Lymphocyte isolation

Fresh blood and saline were mixed in a 1:1 ratio. This mixture was then slowly pipetted over 3 ml of lymphoprep solution (StemCell Technologies) and centrifuged at 600 *g* for 20 min. The white lymphocyte layer obtained was extracted, added to saline and re‐centrifuged at 375 *g* for 10 min. The pellet obtained was incubated in RPMI‐medium at 37°C and 5% CO_2_.

### 2.7 Cell Counting Kit‐8 assay (Cell viability and proliferation assay)

Cell viability and proliferation were evaluated using the cell counting kit‐8 (Abcam). Melanoma and healthy human lymphocytes were treated with 0, 100, 200, 300 and 400 μg/ml of cord blood‐derived exosomes for 24, 48 and 72 h in a 96 well plate and incubated at 37°C. The plates were incubated for 1–4 h, followed by the addition of 1–10 μl of CCK‐8 solution to each plate. The plates were read at 450 nm (Thermo MK3).

### 2.8 Comet assay for determination of DNA damage

Melanoma and healthy human lymphocytes were treated with the optimum exosomal dose of 300 μg/ml for 48 h at 37°C and embedded in 0.5% low melting‐point agarose on 1% agarose‐coated Superfrost™ glass slides. The slides were submerged in a cold lysis solution (100 mM EDTA, 2.5 M NaCl, 10mM Trizma base, 10% DMSO and 1% Triton X‐100 at pH 10). Electrophoresis was performed for 30 min at 25 V (0.8 V/cm), and the current was adjusted to 300 mA. After electrophoresis, neutralizing buffer (400 mM Tris‐HCl, pH 7.5) was added for 5 min, three times. For each slide, 100 cells were randomly scored. The DNA damage was assessed by measuring the % Tail DNA and Olive tail moment (OTM).

### 2.9 RNA isolation and small RNA library construction and sequencing

Following the manufacturer's instructions, total RNA was isolated using the exoRNeasy maxi kit (Qiagen). The isolated RNA was assessed for its quality and quantity using Agilent 2100 Bioanalyzer. The concentrated RNA was later amplified using SenseAMP Plus Low Molecular Weight RNA Amplification Kit (Genisphere, Hatfield). The amplification steps included the generation of poly(A) tails followed by priming the RNA using Oligo (dT) to produce single‐stranded cDNA. The cDNA was refined and tailed dTTP by adding terminal deoxynucleotidyl transferase. Annealing of the 3′ end of the cDNA was carried out with a T7 template. A double‐stranded T7 promoter was generated by the addition of the Klenow enzyme to the 3' end of first‐strand cDNA to yield a small RNA fraction in the range of 18–200 nucleotides. The cDNA was finally generated following the manufacturer's instructions (Illumina) by ligating 5′ and 3′ RNA adapters to small RNA fractions. The resulting strands were then amplified using the sequencing identifier of Illumina. Small RNA genome mapping was achieved using Seqmap. Read maps were performed based on overlapped intersected mappings with known miRNAs.

### 2.10 Statistical analysis

Data are expressed as mean ± standard deviation, and one‐way anova was used to analyse the data between the two groups. Statistical significance was set at a *p*‐value of *p* < 0 0.05(*), *p* < 0.01(**) and *p* < 0.001(***). The analysis was performed using GraphPad Prism 8.0.

## 3 RESULTS

### 3.1 Identification of cord blood‐derived exosomes by SEM

**FIGURE 1:**
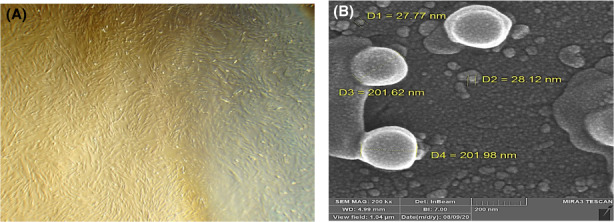
Isolation and morphology of cord blood‐derived exosomes (A) Morphology of Unrestricted somatic stem cells scale bar = 104 μm, (B) SEM imaging of exosomes with 200 KeV magnification.

The cord blood‐derived exosomes showed a typical spherical morphology with diameters ranging from 30 to 120 nm, using a transmission electron microscope at 250 keV (Figure 1). The concentration of the exosomes was observed to be 3.4 × 10^9^ particle/ml.

### 3.2 CBSC ‐derived exosomes caused a significant time and dose‐dependent decrease in the cell survival rate of CHL‐1 cells and lymphocytes obtained from melanoma patients compared to healthy individuals

**FIGURE 2:**
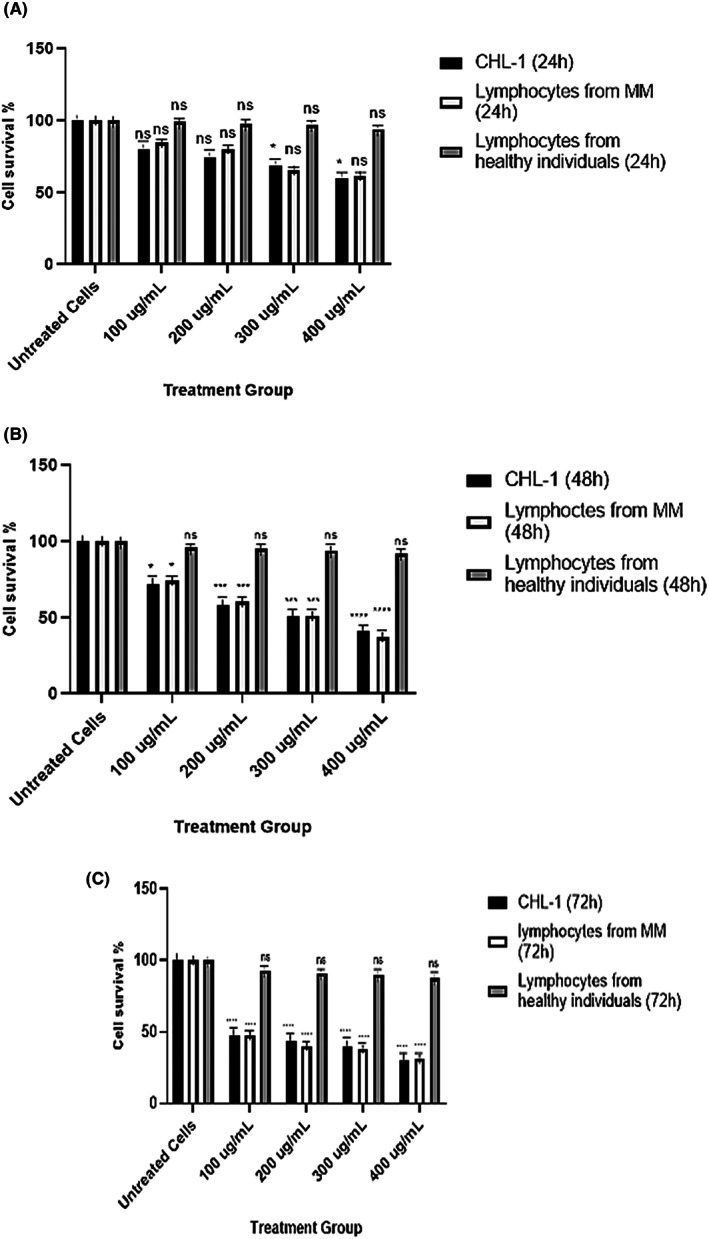
(A–C) Melanoma cells (CHL‐1), lymphocytes from malignant melanoma patients (LMM) and lymphocytes from healthy individuals were incubated separately with various concentrations of 0, 100, 200, 300 and 400 μg/ml protein of cord blood‐derived exosomes for 24, 48 and 72 h, ((A–C) respectively, (*n* = 3)). Cell proliferation analysis was performed by CCK‐8 assay. Errors bars represent SEM. (*p* = ns, *p < 0.05, ***p < 0.001, ****p < 0.0001), data was compared to the untreated cells, analysed by one way anova.

The viability of CHL‐1 cells was assessed by CCK‐8 assay after treatment with varying concentrations of exosomes (0, 100, 200, 300, 400 μg/ml protein) at 24, 48 and 72 h, respectively (Figure 2A–C). The results showed a significant decrease in the viability of CHL‐1 cells treated with exosomes of 100, 200, 300 and 400 μg/ml protein at 48 and 72 h. However, at 24 h, a significant decrease in cell survival percentage was only observed at 300 and 400 μg/ml protein. (*p* = ns, *p* < 0.05, *p* < 0.001, *p* < 0.0001). Similarly, the lymphocytes from melanoma patients displayed a significant time and dose‐dependent decrease in the percentage survival rate of lymphocytes at 48 and 72 h (*p* < 0.05, *p* < 0.001, *p* < 0.0001) (*n* = 3). The overall result contrasted with the high survival rate observed in healthy lymphocytes treated with exosomes as the data shows no significant cytotoxicity of the cord blood‐derived exosomes against the healthy lymphocytes throughout the different incubation periods of 24, 48 or 72 h (*p* = ns) (*n* = 3). By comparing the significant reduction in percentage survival rate at 48 and 72 h; 300 μg/ml protein at 48 h was chosen as the optimal dose with 51% cell viability.

### 3.3 CBSC‐derived exosomes displayed significant genotoxic effects on CHL‐1 cells, lymphocytes from melanoma patients and healthy volunteers

**FIGURE 3:**
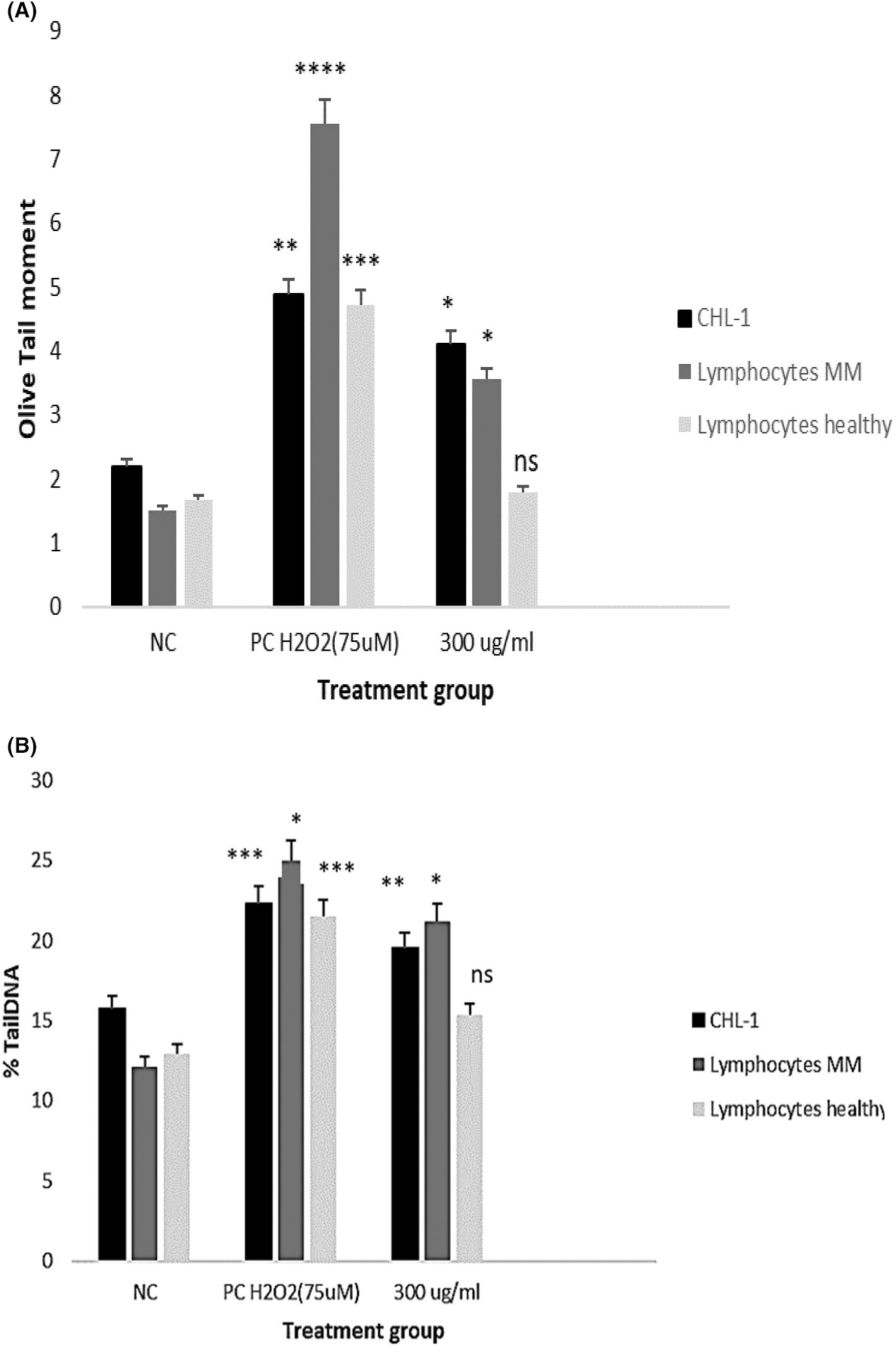
(A, B) OTM and % Tail DNA data show the effect of cord blood‐derived exosomes in Melanoma cells (CHL‐1) lymphocytes from malignant melanoma patients (MM) and lymphocytes from healthy individuals after treatment with an exosomal dose of 300 μg/ml protein. Hundred random cells were counted for each treatment, including negative control (NC), positive control (PC), H_2_O_2_ 75 μM and 300 μg/ml protein of exosomes. All the treatment groups were compared to the NC. Errors bars represent SEM. (*p* = ns, *p < 0.05, **p < 0.01, ***p < 0.001, ****p < 0.0001) (*n* = 3), data were compared to the untreated cells and analysed by one way anova.

To investigate the genotoxic effects of CBSC‐derived exosomes on CHL‐1 cells, lymphocytes from melanoma patients and healthy individuals, a Comet assay was performed. The data obtained are presented as Olive tail moment (OTM) and % tail DNA. The genotoxic effects of CBSC‐ derived exosomes on CHL‐1 cells were analysed. The OTM and %Tail DNA data revealed a significant level of DNA damage caused by exosomes on CHL‐1 cells (*p < 0.05). When compared to the corresponding untreated control, %Tail DNA data showed a significant increase in the level of DNA damage observed on CHL‐1 cells (**p < 0.01). Significant genotoxicity was also observed when the positive control was compared to the negative control of each group (**p < 0.01, ***p < 0.001) (Figure 3A,B).

The OTM data from cancer patients displayed significant levels of DNA damage on melanoma lymphocytes treated with 300 ug/ml of cord blood exosomes at 48 h, compared to the untreated control (*p < 0.05) (Figure 3A,B). A highly significant level of DNA damage was observed when comparing the positive control to the untreated control (****p < 0.0001). The lymphocytes obtained from melanoma patients illustrated significant levels of DNA damage in the exosome treated group, compared to their untreated control (*n* = 3).

Unlike the data from lymphocytes of melanoma patients, the OTM and % tail DNA revealed no significant side effects in healthy lymphocytes after treatment with the optimal concentration of exosomes (300 ug/ml protein) at 48 h, compared to the control group (*p* = ns) However, a significant level of DNA damage was observed when healthy lymphocytes were treated with the positive control (H_2_O_2_ 75 μM) in comparison to the untreated group (***p < 0.001) (*n* = 3) **(**Figure 3A,B).

### 3.4 Next‐generation sequencing of exosomal miRNAs

#### 3.4.1 Assessment of exosomal small RNAs and their length distribution

**FIGURE 4:**
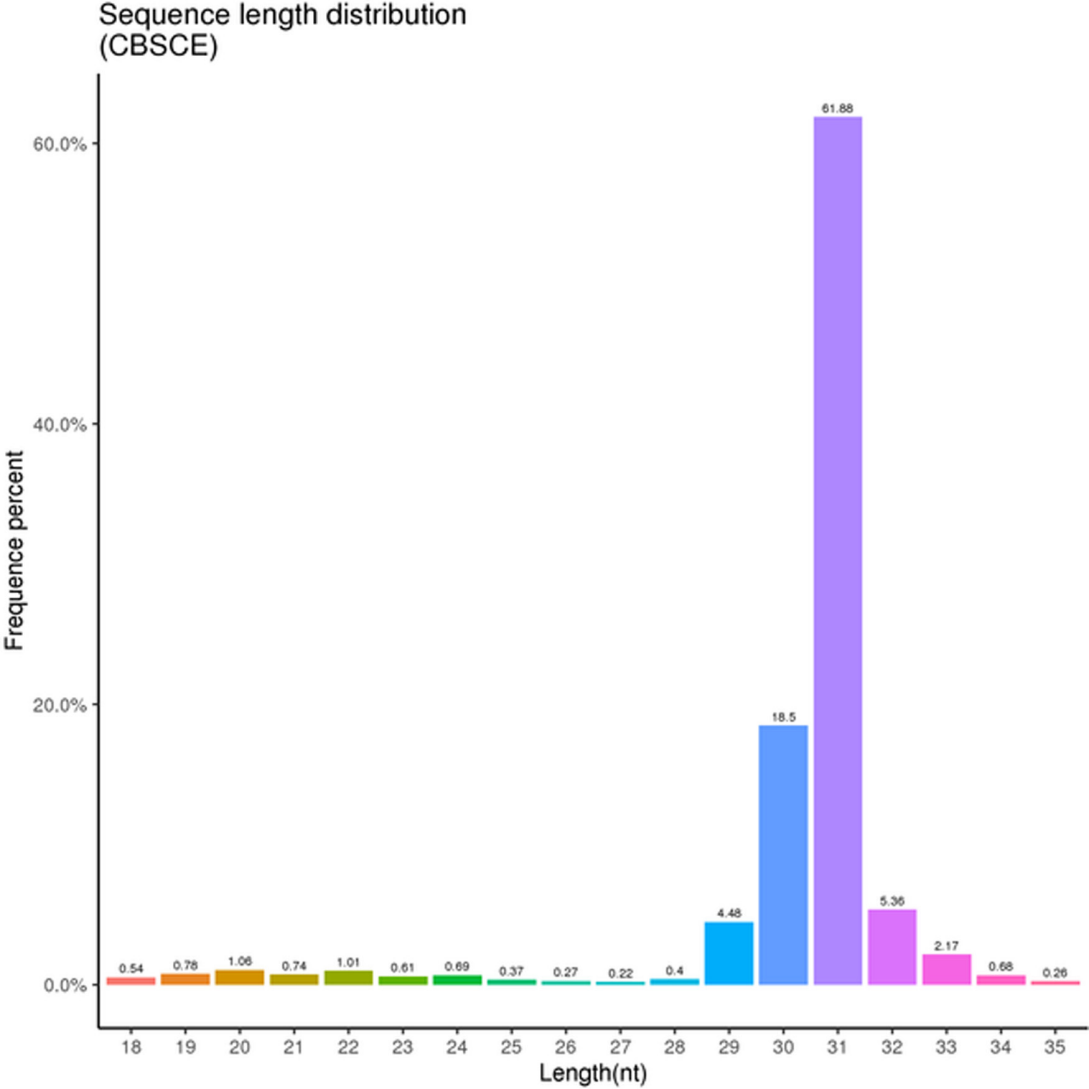
Nucleotide (nt) sequencing and length distribution of small RNAs.

The length distribution of small RNAs ranged from 18 to 40 nucleotides (nt) (Figure 4), with mRNAs usually being 21–22 nt, siRNAs 24 nt and piRNAs between 28–30 nt long.

#### 3.4.2 Sequencing of small RNAs from cord blood exosomes

**FIGURE 5:**
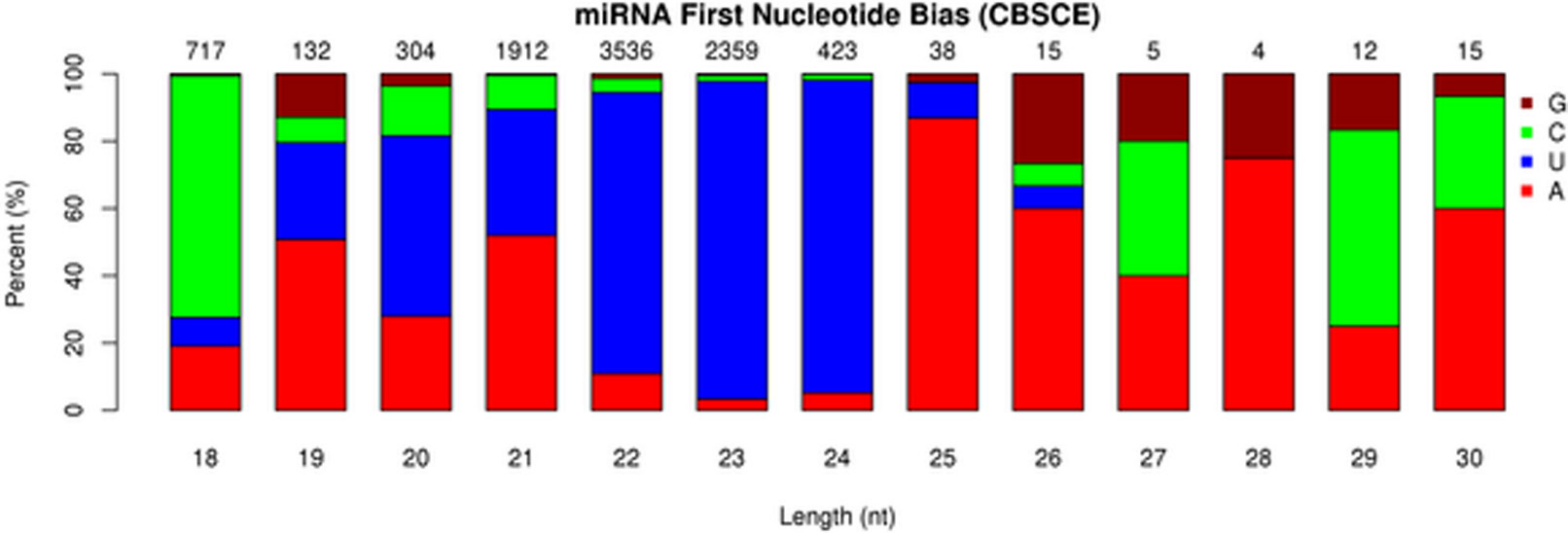
Details of the mapped known miRNAs. The annotation of the miRNA hairpin in each position comprises 5 representing the 5′ region, 3 representing the 3′ region, and f representing other regions of the hairpin, respectively.

Internal sequencing of the cord blood‐derived exosomes was performed extensively to observe their cargo. The cDNA library was analysed on Illumina Genome Analyser, detecting a total of 18,008,025 sequence reads. Sequences were mapped according to the overlaps of the available genome annotations, such as those of miRNAs, rRNAs, tRNAs and other small RNAs. Out of 9481 sRNAs, a total of 270 and 503 known miRNA and sRNAs were detected, respectively. Furthermore, the miRNA expression profile identified the presence of let‐7a‐5p, let‐7b‐5p, let‐7c‐5p, let‐7d‐3p, let‐7d‐5p and 2 novel miRNAs (Figures 1 and 5).

## 4 DISCUSSION

Despite improvements being made in both surgical procedures and cell‐based therapies over the last few years, these methods often come with their own set of potential complications. For example, increased infection risk and toxicity. The use of stem cells could offer a promising alternative to treat tumours and potentially other immune diseases for which the existing therapeutic strategies pose significant challenges. In the present study, we aimed to test whether the cargo of cord blood stem cell‐derived exosomes comprises certain embryonic stem cell reprogramming factors that may be able to inhibit tumour development.

This study tested the potential genotoxic effect of CBSC‐derived exosomes on melanoma (CHL‐1) cells and peripheral lymphocytes obtained from patients with melanoma. Extensive research has shown that assessing DNA damage in surrogate cells, such as peripheral blood lymphocytes of cancer patients, allows researchers to predict how tumour cells would respond to different forms of treatment, whether it be a genotoxic agent or a potential antigenotoxic agent. The results of this study show that treatment with CBSC‐derived exosomes caused a significant time and dose‐dependent decrease in the cell survival of both the cancer cells (CHL‐1 cells) and lymphocytes obtained from melanoma patients. These exosomes were also shown to exhibit significant genotoxicity to cancer cells and lymphocytes obtained from melanoma patients, whereas no significant cytotoxicity was observed in the lymphocytes of healthy volunteers.

It is reported that exosomes play a significant role in maintaining the healthy physiology of the cells.^28^ They are involved in essential processes in multicellular organisms, such as cell signalling and intercellular communication. Exosomes are released by different types of healthy cells, such as epithelial cells, adipocytes, Schwann cells, neurons and haematopoietic cells. In addition, these nanovesicles are also present in different types of biological fluids, including breast milk, urine, sperm, saliva, tears, cerebrospinal fluid, amniotic fluid and synovial fluid.^28^ Exosomes play an immense role in regulating normal physiological functions in normal healthy tissues. For instance, in the brain, these nanovesicles potentiate the integrity of axons, microglia and myelination. Maintaining the integrity of axons is done through certain enzymes, such as catalase and superoxide dismutase‐1, that resist oxidative stress. This study suggested that exosomes released from oligodendrocytes increase the stress tolerance of neurons.^29^ Furthermore, exosomes are also considered to play a significant role in carrying out the normal physiological processes of the cardiovascular system. Under hypoxic conditions, the cardiomyocytes release exosomes by secreting TNF‐α to induce apoptosis. Hence, this study revealed that under stressful conditions, exosomes are able to propagate inflammatory responses.^30^

Extensive research has shown that exosomes exert adverse effects on tumour growth. These negative effects depend on various conditions, including the tumour type, the stage of development and the expression of tumour suppressor molecules. Exosomes have been shown to express and deliver anticarcinogenic molecules that display tumour suppressor activities in recipient cells. Consequently, they can inhibit tumour growth by targeting a variety of signalling pathways, including angiogenic and growth regulatory pathways. For example, exosomes from bone marrow‐derived MSC (BM‐MSCs) have been shown to act as negative cell cycle regulators and exhibit inhibitory effects on tumour growth. Additionally, BM‐MSCs‐derived exosomes can transfer miRNAs from the bone marrow, ultimately promoting tumour dormancy in metastatic breast cancer. Moreover, a recent study showed that MSC‐derived exosomes could inhibit breast cancer via miRNA‐mediated VEGF suppression.

In the present study, the CBSC‐derived exosomes were postulated to inhibit the proliferation of peripheral lymphocytes obtained from patients with melanoma and CHL‐1 cancer cells by inducing apoptosis and lowering their tumorigenicity in vitro. This was speculated to be via the expression of the *let‐7* miRNA family (let‐7a‐5p, let‐7b‐5p, let‐7c‐5p, let‐7d‐3p and let‐7d‐5p) that was obtained by the internal sequencing of the cord blood exosomes (Table 1). Research suggests that several types of miRNAs exist in different carcinomas. Some act on the expression of oncogenes that target the tumour suppressor genes, and their overexpression results in cancer cell proliferation, such as the miR‐183. Alternatively, the other types, such as the miR‐10,016, may impact the expression of tumour suppressor genes in reducing cancer cell migration.^18^ Similar to the miR‐100, the miRNA let‐7 family also functions as cancer suppressor genes by inhibiting cancer cell proliferation via suppressing the expression of HMGA2, inducing apoptosis, and preventing cancer cell migration.^31^ The apoptotic effects of let‐7 miRNA have been extensively investigated in various tumours. In hepatocellular carcinoma, the presence of let‐7a‐5p is speculated to result in BCL2L1 suppression, ultimately resulting in apoptosis of cancerous hepatocytes.^32^ Similarly, another study suggested that the overexpression of let‐7b‐5p is associated with inhibiting multiple myeloma. This study concluded that let‐7a‐5p immensely downregulated cancer cell proliferation and induced apoptosis of RPMI‐8226 cells.^33^ These findings are consistent with the present data as a decrease in cell survival was observed in both lymphocytes obtained from patients with melanoma and CHL‐1 cancer cells, indicating an increase in apoptosis.

**TABLE 1:** Known miRNA expression profile of CBSC‐derived exosomes.

miRNA	CBSCE
hsa‐let‐7a‐5p	145.00
hsa‐let‐7b‐5p	76.00
hsa‐let‐7c‐5p	33.00
hsa‐let‐7d‐3p	8.00
hsa‐let‐7d‐5p	8.00

Additionally, the significance of miR‐let‐7c‐5p expression in the regulation of tumour development has recently been highlighted. In bladder cancer, the expression of miR‐let‐7c‐5p is reported to be downregulated, thus increasing HMGA2 expression significantly. This study suggests that the upregulation of miR‐let‐7c‐5p inhibits bladder cancer progression by targeting *HMGA2* protein.^34^ Furthermore, another study investigated the effectiveness of miR‐ let‐7d‐3p in the apoptosis of ovarian epithelial cells and their sensitization to chemotherapeutics. Cellular death induction and positive response to chemotherapy agents such as carboplatin/paclitaxel were observed by this study in the presence of an overly expressed let‐7d‐3p miRNA.^35^ Research suggests that the miRNA let‐7 family possesses the ability to concurrently induce cellular death and inhibit cancer progression in several aggressive carcinomas. This was consistent with our results. However, interestingly, we also observed this effect in melanoma tumour environment‐exposed lymphocytes. These miRNAs are also depicted to restore chemosensitivity in certain resistant tumours. In ovarian cancer, the expression of let‐7d‐5p miRNA upregulated the *p53* signalling pathway via silencing the HMGA1 expression, subsequently leading to a better prognosis.^36^

Based on this investigation, it was concluded that cord blood‐derived exosomes possess the ability to prevent tumour induction by exhibiting certain miRNAs that might prove beneficial in reducing cancer cell tumorigenicity and growth. The results of this study are consistent with the already established research that highlights the anticarcinogenic effects of the miRNA let‐7 family, namely, let‐7a‐5p, let‐7b‐5p, let‐7c‐5p, let‐7d‐3p and let‐7d‐5p, respectively. The two novel miRNAs are the main subject for more investigation into modifying the cancer cells' function. Research is currently underway to understand the exact mechanisms of these miRNA subtypes in multiple types of cancer. The data of this investigation implicates a better understanding of exosome behaviour in treating multiple types of invasive carcinomas.

## AUTHOR CONTRIBUTIONS

**Parisa Naeem:** Investigation (lead); writing – original draft (lead). **Adi Baumgartner:** Supervision (supporting); validation (supporting); writing – review and editing (lead). **Nader Ghaderi:** Data curation (supporting); investigation (supporting); resources (supporting); supervision (supporting). **Farshid Sefat:** Funding acquisition (supporting); resources (supporting). **Maysa Alhawamdeh:** Investigation (supporting); methodology (supporting). **Pouria Akhbari:** Formal analysis (supporting); validation (supporting); writing – review and editing (supporting). **Karthic Swaminathan:** Methodology (supporting); resources (supporting). **Saeed Heidari:** Methodology (supporting); resources (supporting). **Diana Anderson:** Supervision (supporting). **Andrew Wright:** Investigation (supporting); methodology (supporting); supervision (supporting). **Mohammad Isreb:** Supervision (supporting). **Mojgan Najafzadeh:** Conceptualization (lead); investigation (lead); methodology (lead); project administration (lead); supervision (lead); validation (lead). **Fanila Shahzad:** Writing – review and editing (supporting).

## CONFLICT OF INTEREST

Mojgan Najafzadeh: I declare that there is no conflict of interest in relation to this paper.

## Data Availability

Data available on request due to privacy/ethical restrictions. The data that support the findings of this study are available on request from the corresponding author. The data are not publicly available due to privacy or ethical restrictions.
